# Estimation of Bone Trace Elements Following Prolonged Every-other Day Feeding in C57BL/6 Male and Female Mice

**DOI:** 10.1007/s12011-021-02875-z

**Published:** 2021-08-20

**Authors:** Katarzyna Zgutka, Katarzyna Piotrowska, Mateusz Bosiacki, Andrzej Pawlik, Maciej Tarnowski

**Affiliations:** 1 Department of Physiology, Pomeranian Medical University in Szczecin, 72 Powstancow Wlkp Street, 70-111 Szczecin, Poland; 2 Department of Functional Diagnostics and Physical Medicine, Pomeranian Medical University in Szczecin, 54 Żołnierska Str, 71-210 Szczecin, Poland

**Keywords:** Diet, Trace elements, Bone health, Every-other day feeding, Dietary interventions

## Abstract

The purpose of this study was to examine the effect of prolonged every-other day (EOD) feeding on bone trace elements. Four-week old C57BL/6 female (*n* = 12) and male (*n* = 12) mice were employed in this experiment. Animals were assigned to four groups: ad libitum—AL (males and females), EOD fed (males, females). After 9 months, the mice were sacrificed. Long bones (humerus and radius) were isolated and prepared for analysis using inductively coupled plasma optical emission spectrometry to determine the Fe, Zn, Mo, Co, Cu, Mn, Cr contents. Estimation of cathepsin K expression on bone slides was performed to determine the activity of osteoclasts in bones of EOD- and AL-fed animals. Higher content of Fe in EOD-fed females compared to AL-fed females was found. In EOD-fed males, a significantly higher amount of Mo (*p* < 0.005) and Co (*p* < 0.05) in comparison to AL-fed males was noted. Gender differences in amounts of trace elements in control AL-fed males vs. females were observed. EOD feeding influences the amount of some trace elements in long bones of female and male C57BL/6 mice. However, this is not influenced by the activity of bone cells.

## Introduction

Among environmental factors, diet is critical for bone growth and loss. Proper bone development and maintenance requires adequate composition of nutrients containing amino acids, energy resources, hydroxyapatite forming (calcium, Ca and phosphorus, P) and trace elements [1–3]. Trace elements are dietary elements that occur in very small amounts (0.01%) relative to the mass of the organism. The World Health Organisation (WHO) subdivided 19 trace elements into three main groups: (i) essential elements of which an imbalance may be considered a risk factor for diseases important for public health (zinc, Zn; chromium, Cr; cobalt, Co; selenium, Se; molybdenum, Mo; iodine, I); (ii) probably essential (manganese, Mn); (iii) potentially toxic (fluorine, F; lead, Pb; cadmium. Cd; mercury, Hg; lit, Li) [1]. Some of these trace elements are also essential for skeletal development and growth, and bone homeostasis. Zinc, copper and manganese have osteoprotective effects, while cadmium and cobalt, if overdosed, are toxic for bone cells [1, 4, 5].

An adequate intake of trace elements is required for proper development of the skeleton in childhood and adolescence. Rodents have been used to study various aspects of bone formation and loss. In rodents, as in humans, bone formation occurs rapidly during early post-natal development (in mice, to at least 2 months of age) [6]. Malnutrition or under nutrition in these periods may lead to linear growth retardation, poor bone and cartilage quality and function [7–9]. Some adults may also be prone to a dietary deficiency of trace elements: people exposed to physical and psychological stress, including athletes and those who work in leadership positions, and the elderly [4, 9]. In these individuals, the risk of osteoporotic fractures has been noted [4]. Dietary deficiency may also develop in individuals using restrictive or eliminative diets.

Previous studies have demonstrated that different dietary modifications contribute to levels of individual trace elements in bones. Every-other day feeding (EOD) is one form of caloric restriction and beneficial effects of this feeding regime are well described [10, 11]. Xie et al. showed that intermittent periods of fasting achieved through EOD protect mice against neoplastic disease but do not delay aging in these animals [11]. The lack of intake of trace elements in this type of feeding regime during fasting days is not supplemented during feeding days although, some authors noted increased food consumption in EOD-fed animals during feeding days [12]. Our data show that, during long-term experiments, cumulative food intake was decreased in EOD-fed mice in comparison to ad libitum fed animals. Previous analysis of calcium (Ca), phosphorus (P), potassium (K), magnesium (Mg) and sodium (Na) did not show significant changes in mineral composition between ad libitum and EOD fed males and females [13]. Our goal was to establish if prolonged EOD feeding influences the amount of trace elements in long bones of female and male C57BL/6 mice.

## Materials and Methods

### Animals and Diet

Four-week-old C57BL/6 female (*n* = 12) and male (*n* = 12) mice were employed in this experiment. The mice were housed separately in cages (one mouse per cage) and kept in a 12-h light/12-h dark cycle, under controlled a temperature (21 °C) and ventilation. Animals of both genders were assigned to four groups: ad libitum—AL (males and females), EOD-fed (males, females) ad libitum during feeding days and deprived of food during fasting days, tap water was provided ad libitum to all animals. The EOD feeding model was chosen due to previous reports confirming its effect on prolonging life expectancy in rodents [11]. All animals were fed with Labofeed H (60% carbohydrate, 30% protein, 10% fat) (Morawski, Poland). Trace elements content in Labofeed H chow was Fe 150.0 mg, Mn 30 mg, Zn 50 mg, Cu 12 mg per 1 kg of chow. Other mineral ingredients which were analysed in this study were not supplemented**.** Food was given and removed at 5 p.m. To evaluate the amount of food consumed, it was weighed just before the removal. The Labofeed H chow came in solid pellets. Animals did not crumble these pellets and during the days of the restriction it was enough to remove the chow without replacing the cage. Body weight was measured weekly during the time of the study.

After 9 months, the mice were sacrificed. Long bones (humerus and radius) were taken and prepared for further analysis. All animal protocols were approved by the Local Ethical Committee (approval no 27/2012).

### Bone Trace Element Content Analysis

All samples were cleaned of excess flesh, tendons and ligaments, transferred into 1.5-ml microtubes and stored at − 80 °C until processed. Samples were analysed using inductively coupled plasma optical emission spectrometry (ICP-OES, ICAP 7400 Duo, Thermo Scientific) equipped with a concentric nebuliser and cyclonic spray chamber to determine the Fe, Zn, Mo, Co, Cu, Mn, Cr contents. Analysis was performed in radial mode. Samples were thawed at room temperature and dried overnight at 70 °C to a constant weight after cleaning of all adherent tissue. Bones were ground into powder in a porcelain mortar and mineralised using a microwave digestion system, MARS 5, CEM. The weight of the bone tissue used for analysis was at least 0.052 g. Samples were transferred to clean polypropylene tubes, where 1 ml of 65% HNO_3_ (Suprapur, Merck) was added to each vial and each sample was allowed 30 min pre-reaction time in a clean hood. After completion of the pre-reaction time, 1 ml of non-stabilised 30% H_2_O_2_ solution (Suprapur, Merck) was added to each vial. Once the addition of all reagents was complete, samples were placed in special Teflon vessels and heated in a microwave digestion system for 35 min at 180 °C (15 min ramp to 180 °C and maintained at 180 °C for 20 min). At the end of digestion, all samples were removed from the microwave and allowed to cool to room temperature. In the clean hood, samples were transferred to acid-washed 15 ml polypropylene sample tubes. A further 100-fold and 25-fold dilution was performed prior to ICP-OES measurement. A volume of 100 µl was taken from each digest. Samples were spiked with an internal standard to provide a final concentration of 0.5 mg/L Ytrium in 1 ml of 1% Triton (Triton X-100, Sigma) and diluted to a final volume of 10 ml with 0.075% nitric acid (Suprapur, Merck). Samples were stored in a monitored refrigerator at a nominal temperature of 8 °C until analysis. Blank samples were prepared by adding concentrated nitric acid (50 µl) to tubes without a sample and subsequently diluted in the same manner as described above. Blank samples were analysed at the beginning of the analysis and after every 10 samples. Multi-element calibration standards (ICP multi-element standard solution IV, Merck for Zn, Cu, Mn, Fe, Cr, Co; ICAP 6000 Multi-Element Test Solution) were prepared with different concentrations of inorganic elements in the same manner as in blanks and samples.

Deionised water (Direct Q UV, Millipore, approximately 18.0 MΩ) was used for the preparation of all solutions. The wavelengths (nm) were 206.200 (Zn), 224.700 (Cu), 257.610 (Mn), 259.940 (Fe), 205.560 (Cr), 228.616 (Co), 204.598 (Mo).

Validation was performed by evaluating NIST SRM 8414 and NIST SRM 1486 reference material (National Institute of Standards and Technology, USA) and the recovery of internal standard (yttrium) (Table 1).

**Table 1:** The analysis of NIST SRM 8414 and NIST SRM 1486 reference material (National Institute of Standards and Technology, USA) by ICP-OES

SRM NIST 1486* NIST SRM 8414**			
Chemical elements	Certified	Measured *n* = 6	Recovery (%)
Fe*	99 ± 8	101.870	102.9
Zn*	147 ± 16	138.920	94.5
Mo**	0.08 ± 0.06	0.130	162.5
Co**	0.007 ± 0.003	0.009	128.6
Cu**	2.84 ± 0.45	2.980	104.9
Mn**	0.37 ± 0.09	0.450	121.6
Cr**	0.071 ± 0.038	0.103	145.1

### Immunohistochemical Analysis of Cathepsin K

Deparaffinised sections of bones (3-μm thick) were hydrated and heat epitope retrieval was performed in a microwave oven in a retrieval solution buffer pH = 6 (DAKO, Denmark). After cooling to room temperature (RT), the slides washed with phosphate-buffered saline (PBS) and endogenous peroxidase were blocked with vector blocking solution (Vector Laboratories, USA), after washing with PBS, the slides were further incubated with 2% horse serum (Vector Laboratories, USA). After incubation with serum, slides were incubated with primary antibody: cathepsin K (Santa Cruz Biotech, USA) for 1 h at RT and after double washing in PBS, the slides were incubated with ImmPRESS Universal Antibody Polymer Reagent (ImmPRESS® HRP Universal PLUS Polymer Kit, Peroxidase, Vector Laboratories, USA), after washing in PBS, the reaction was visualised with ImmPACT DAB EqV Substrate(ImmPRESS® HRP Universal PLUS Polymer Kit, Peroxidase, Vector Laboratories, USA). After visualisation, slides were counterstained with haematoxylin (Harris modified haematoxylin, Sigma) and mounted in Canada balsam (all purchased from Sigma-Aldrich, USA) mounting medium and evaluated under an Olympus IX81 inverted microscope (Olympus, Germany). Micrographs were collected with CellSens software (Olympus, Germany).

Statistical Analyses.

Statistical analyses were performed with STATISTICA (StatSoft Poland). A non-parametric *U* Mann–Whitney test was used for small sample sizes. A value of *p* < 0.05 was considered significant.

## Results

### Anlysis of Food Consumption During the Study

**Fig. 1:**
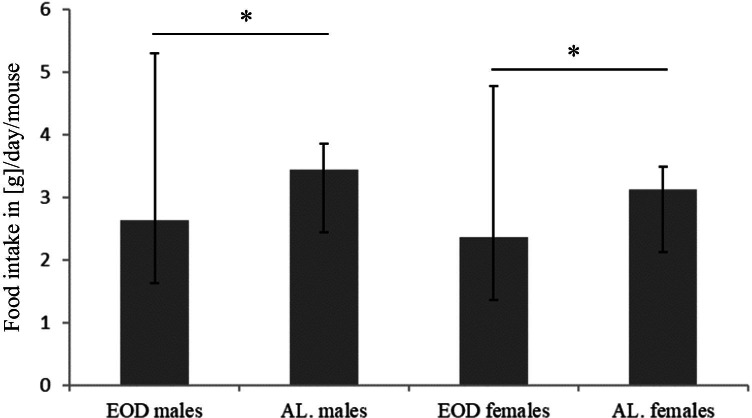
Comparison of food consumption [g]/day/mouse in EOD vs. AL animals both genders *p < 0.01

The recommended amount of Labofeed H chaw per day was 4–5 g per day per mouse. In feeding day food consumption ranged from 4.98 g/day to 5.64 g/day for individual male mice in EOD group: 2.0–5.0 g/day/mice in AL male group. In case of females group: 3.46–5.14 g/day/mouse in EOD and from 2.0 to 4.2 g/day/mouse in AL. However, taking into account the days of fasting, the average food consumption in the EOD group was lower than in the AL groups (Fig. 1).

### Trace Elements Content

We measured the amount of Zn, Cu, Mn, Fe, Cr, Co, Mo in long bones of C57BL/6 mice. We found a higher content of Zn, Fe, Mo, Cu, Mn in EOD-fed females compared to AL-fed females, but only in the case of Fe was the result statistically significant (*p* < 0.05). The content of Co was insignificantly lower in EOD-fed females vs. AL-fed females (Table 2).

**Table 2:** Trace elements in long bones of female mice. The concentrations of elements were expressed as g/kg dry mass (dm) of bones. *p < 0.05

Trace element	AL-fed females	EOD-fed females
	Average ± SD	Average ± SD
Fe	194.754 ± 62.14	274.597 ± 84.96 *
Zn	286.186 ± 64.579	314.691 ± 32.844
Mo	94.824 ± 20.162	96.944 ± 22.020
Co	40.108 ± 9.711	36.374 ± 9.832
Cu	9.596 ± 7.096	10.297 ± 4.736
Mn	10.896 ± 1.212	12.554 ± 2.881
Cr	4.868 ± 1.102	4.933 ± 0.622

In EOD-fed males, we noted a significantly higher amount of Mo (*p* < 0.005) and Co (*p* < 0.05) in comparison to AL-fed males. The higher amount of Fe and Zn in bones of EOD-fed males vs. AL-fed males was insignificant. Cu and Mn were insignificantly lower in bones of EOD-fed males in comparison to AL-fed male mice (Table 3).

**Table 3:** Trace elements in long bones of male mice; the concentrations of elements were expressed as g/kg dry mass (dm) of bones.*p < 0.05, **p < 0.005

Trace element	AL-fed males	EOD-fed males
	Average ± SD	Average ± SD
Fe	262.153 ± 54.97	296.79 ± 189.777
Zn	433.872 ± 87.088	428.24 ± 113.848
Mo	76.065 ± 12.973	114.21 ± 18.573 **
Co	34.862 ± 11.616	44.677 ± 12.933*
Cu	7.548 ± 1.911	6.045 ± 3.613
Mn	13.327 ± 6.158	12.34 ± 1.229
Cr	7.388 ± 1.575	6.98 ± 1.343

We also compared the amounts of trace elements in control (AL-fed) males vs. females to determine if there were any gender differences in trace element content in long bones (Table 4). We observed a lower amount of Mo, Co, Cu in males vs. females and a higher amount of Fe, Zn, Cr, Mn in AL-fed males in comparison to AL-fed females. The results for Fe, Zn, Mo, Co, and Cr were statistically significant (*p < 0.05 and **p < 0.005). We estimated differences in trace elements also in EOD animals (males vs. females). We observed statistically different amounts of Fe, Zn, Mo, Co and Cr (Table 5), but after prolonged EOD we observed reversed relation in Fe and Mo and Co (Table 5).

**Table 4:** Comparison of trace elements in control (AL-fed) mice, males vs. females. *p < 0.05, **p < 0.005

Trace element	AL-fed males	AL-fed females
Fe	134.6% *	100%
Zn	151.6% *	100%
Mo	80.2% *	100%
Co	86.9% *	100%
Cu	78.6%	100%
Mn	122.3%	100%
Cr	151.7% **	100%

**Table 5:** Comparison of trace elements in EOD mice, males vs. females. *p < 0.001, **p < 0.02, ***p < 0.05

Trace element	EOD males	EOD females
Fe	76.43%**	100%
Zn	136.1% ***	100%
Mo	117.8% ***	100%
Co	122.8% ***	100%
Cu	58.7%**	100%
Mn	98.3%	100%
Cr	141.6% *	100%

### Cathepsin K Visualisation on Bone Slides

**Fig. 2:**
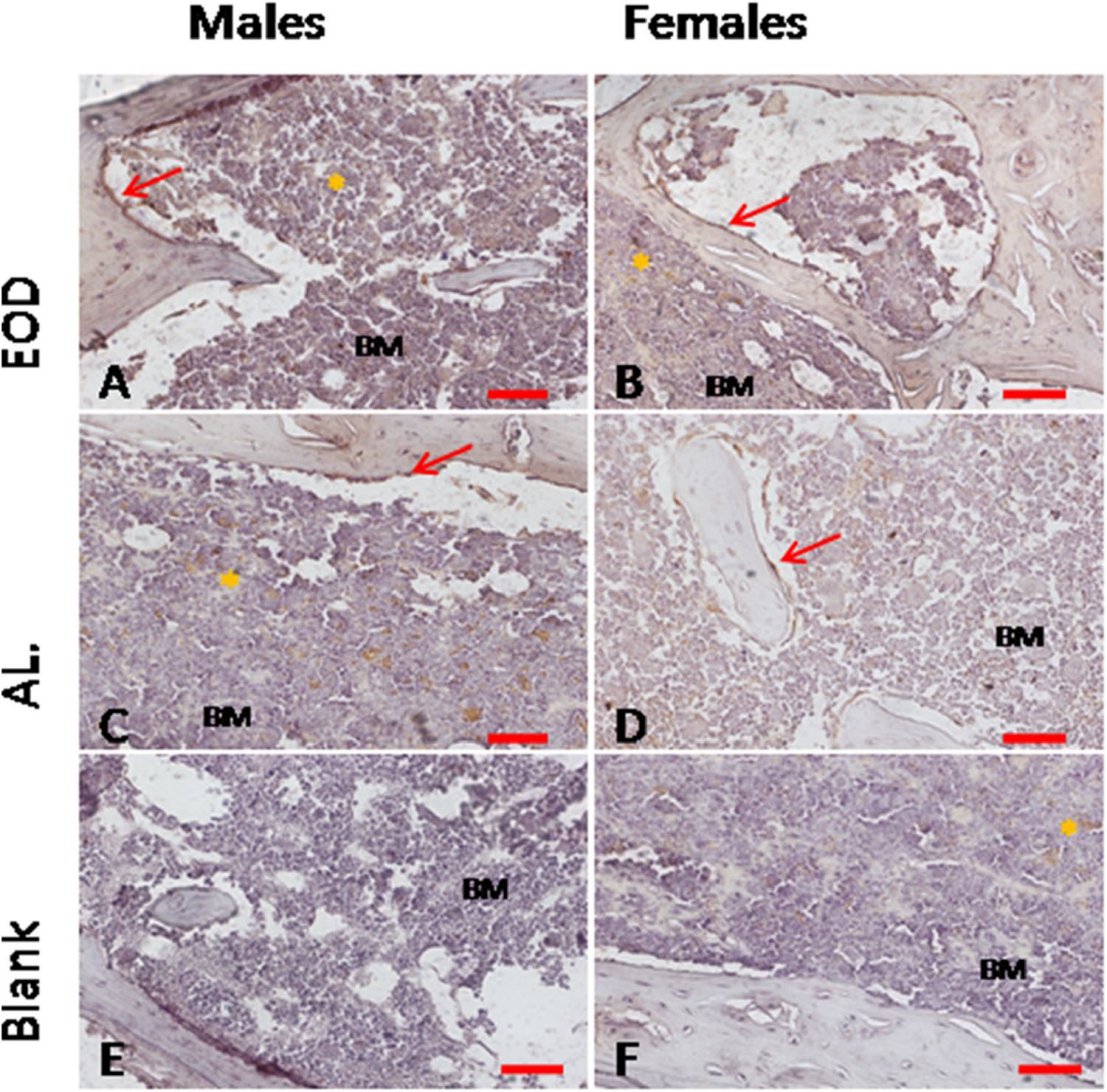
Cathepsin K expression in osteoclasts in long bones of mice. AL, Ad libitum fed mice panels; EOD, every-other day fed mice; BM, bone marrow; males—panels A,C,E; females—panels B,D,F; panels E,F negative controls—sections stained without primary antibody. Asterix—macrophages loaded with hemosiderrin; red arrows—osteoclasts. Scale bar 50 μm

The proper morphology of bones was noted in all studied groups. Parallel collagen fibres were observed and red bone marrow in bone cavities. In some slides of EOD-fed female mice, adipose cells in bone marrow were noted. We performed a visualisation of cathepsin K expression on bone slides to determine the number and changes in morphology due to activity status of osteoclasts in bones of EOD- and AL-fed animals. We observed brown-stained active osteoclasts in bone lacunae pointed with red arrows, but we found no differences in cathepsin K expression in any studied group (Fig. 2). We also noted high number of BM macrophages loaded with hemosiderrin, pointed with asterix.

## Discussion

Bone strength and integrity depend not only on the presence of calcium and phosphorus but also trace elements. Deficiencies in trace elements may lead to growth retardation or osteopenia and osteoporosis [14]. In bones, they have a role in the production of collagen, mineralisation and bone resorption [2, 3, 9].

Zinc (Zn) is one of most essential trace elements accumulated in the skeleton [9]. Zinc is part of many enzymatic systems responsible for cellular growth, proliferation and metabolism [15]. In bones, Zn increases osteoblast activity and collagen formation, inhibits osteoclastic bone resorption and enhances fracture healing [3, 4]. In children, Zn deficiency leads to a decrease of IGF-1 level, which is crucial for skeletal growth [3]. In vitro Zn increases proliferation of cells, differentiation of mesenchymal stem cells (MSC) to osteoblasts, increases mRNA levels for osteogenic genes like alkaline phosphatase, collagen type I and osteocalcin [5]. Zinc supplementation restores a balance in trace element concentrations in blood after cadmium exposition [16–18]. In our study on middle-aged mice, the Zn level in bones was not affected by the EOD diet in either gender. This indicates that restriction in the amount of food intake, without a change in composition of macro- and micronutrients, had no impact on Zn accumulation in murine bones.

Another metal essential for collagen synthesis is iron (Fe). Cross-linking of collagen depends on prolyl hydroxylase containing Fe. In Fe-deficient patients, bone strength decreases and increased porosity of bones is observed [1]. In growing boys, the Fe level in blood (cellular and serum ferritin) is positively correlated with bone mineral content [19]. In adults, overload with iron may lead to enhanced osteoclast formation and activation, which results in enhanced bone resorption [20, 21]. In ovariectomised female mice, low oestradiol status and iron overload caused increased bone resorption through activation of reactive oxygen species (ROS) production and osteoclast activation [22]. In our study, we have shown a significantly higher Fe accumulation in EOD-treated females in comparison to AL-fed females, although we did not observe differences in calcium and phosphorus accumulation in females regardless of the feeding regime [13]. We also revealed a statistically higher content of Fe in bones of males in comparison to females in the AL-fed group, which may indicate a gender difference in the content of this element in bones. To test, if increased iron level in bones has any influence on bone loss and osteoclast activity we performed staining for cathepsin K a well-known osteoclas marker on bone slides [23]. Estimation of active osteoclasts (cathepsin K evaluation) on bone slides showed no differences in cathepsin K expression in either males or females regardless of the studied group. The lack of increased bone loss in EOD-fed females may be a result of the sustained reproductive potential of EOD-fed females, which was indicated by the presence of ovarian follicles in EOD-treated mice ovaries in our previous report [24].

Cross-linking of collagen in bones also depends on the action of lysyl oxidase containing copper (Cu) [1]. This metal prevents osteoclast activation by decreasing the amount of ROS and prevents osteoblast apoptosis [1, 3]. In experiments with bone injuries, implants containing Cu increased bone healing by increasing osteoblast proliferation, vascularisation of the injured area and osteoprogenitor recruitment [25]. In vitro studies with Cu-covered scaffolds showed increased compact proliferation, increased cell to cell interaction and cell to matrix interaction [26]. Moreover, Cu decreased the number of osteoclasts or decreased their activity in bone cementum, used for bone healing [27]. In our study, Cu content varied insignificantly between groups. In adult rodents, the amount of Cu in various tissues is constant [28]. Moreover, Cu absorption depends on Zn absorption because of competition for absorption sites [2]. The insignificant changes of Zn found in our study correspond with similar changes in Cu accumulation, which may reflect an unchanged balance between these two metals during prolonged EOD feeding.

Another metal found in enzyme active sites and accumulated in bones is molybdenum (Mo) [2, 9]. In experiments with Cr-Co-Mo scaffolds, an increased number of osteogenic cells was found [29]. In our study, we found a significantly higher accumulation of Mo in bones of EOD-fed males vs. AL-fed males. Mo absorption in the gut reaches 88–93% of intake in young men [30]. Turnover of Mo is very slow when the intake of this metal is very low [30]. The low turnover of Mo during fasting days may explain the increased accumulation of Mo during the long experiment. The lack of a difference in accumulation of this trace element in females may indicate a gender difference in the rate of turnover of Mo, as we also noted a significantly lower amount of Mo in control (AL-fed) females in comparison to control males.

The bones are important deposit site for manganese (Mn) and about 25–40% of this metal is stored in the skeleton [9]. Mn is essential as a cofactor in many enzymatic systems, but excessive Mn may be toxic for the nervous system [31]. In the skeleton, Mn favours bone formation, enhances trabeculae thickness and increases their number [32]. In our study, the amount of Mn in long bones varied insignificantly between the groups, which may be the result of changeable Mn absorption in the gut that prevents overdosing and previously reported stable accumulation levels of this metal in rat’s tissues [28, 31].

In the present study, we also established the amount of cobalt (Co) and chromium (Cr). In the majority, Co is ingested in the form of vitamin B12 and in normal conditions 70% of B12 is absorbed in the gut [33]. Cobalt primarily accumulates in the liver but the skeleton is also an important site for storage of this metal [9]. After administration in large doses, i.e. in industry, it may be genotoxic [9]. In vitro studies have revealed that cultivating osteoclasts with Co-containing cementum increases cell activity [27]. In our study with prolonged EOD feeding, we observed a significantly higher accumulation of Co in bones of male mice after EOD treatment. We also noted a significantly higher accumulation of Co in bones of control females in comparison to control males. This is consistent with data obtained from human patients with a higher concentration of this metal in urine of women [33]. This may indicate a gender specific turnover of this metal and response to EOD treatment.

Chromium controls blood sugar level and adipose tissue level [34]. It also affects bones through oxidative stress [32]. In low dosages, Cr causes osteoclast formation and resorption of bone [27]. The EOD feeding did not influence the accumulation of Cr in long bones, which is consistent with other studies showing a balance between intake and excretion of this potentially harmful metal [35].

Although we observed a minor influence of prolonged food restriction on the accumulation of Zn, Mn, Cr, Cu in bones of female and male mice, we observed significant differences in accumulation in control groups: males vs. females. These differences include Fe, Zn, Mo, Co, Cr amounts in long bones of middle-aged mice. Gender differences in trace element content were described previously for different tissues [28, 33, 36–38]. This indicates that pathways for trace element absorption and/or excretion vary between genders. We also noted a different response to EOD feeding in males and females in the accumulation of trace elements, consistent with previous findings concerning gender differences in response to caloric restriction in various tissues [13, 39, 40].

There are few different dietary regiments used in research. Calorie restriction in every day feeding is commonly used method where amount of calories is decreased and varies between 15 and 60% reduction of baseline needs [41]. Other protocols use fasting as method of decrease in calorie intake. EOD or alternate day fasting (ADF) are used in human and animal studies on benefits of dietary restrictions. Both, CR and EOD/ADF leading to weight loss, lifespan increase, other health benefits (decreased glucose, cholesterol, blood pressure) [11, 41]. Apart from evident increase in lifespan and other health benefits EOD diet is not very restrictive. In EOD, average calorie intake is only 7.8% lower in mice in comparison to AL feeding [11]. In our study, average calorie intake was 23.5% lower than in the AL group. Even in long periods of treatment, there are no evident symptoms of malnutrition and examination of body composition of EOD animals revealed increased amount of adipose tissue [11].

In this paper, we showed the influence of EOD feeding on bones trace elements content. Only Fe in females and Mo and Co in males were significantly higher in EOD mice in comparison to AL-fed animals. In our previous study, we noted no significant changes in Ca, P and Mg during prolonged EOD treatment in mice [40]. That indicates lack of significant change in mineral balance during lifelong EOD. We can speculate, that as a single indicator EOD treatment is not increasing fracture risk. It is in compliance with other studies in rodents and humans, where EOD did not influenced structural and biomechanical properties of bones [11, 42, 43]. Although Fe, Co are elements responsible for osteoclasts formation and activation, we noted no increase in osteoclast amount and activity. It seems that increase of Fe in females and Mo and Co in males as a single factor is not sufficient for activation of massive bone resorption. The reasons for trace metal accumulation may be changes in metabolism, water turnover and amelioration of age-dependent pathologies in organs (i.e. kidneys) during lifelong EOD treatment observed in mice [11]. The limitation of this study is lack of bone mineral density estimation as we were limited by number of animals used in the study. Other issue which may be considered as limitation is lack of tests establishing trace elements absorption and excretion as these issues were not primary goal of experiment with calorie restriction and aging performed by our research group.

## Conclusion

Every-other day feeding (EOD), which is one form of caloric restriction, contribute to levels of some individual trace elements in bones. However, the differences which were observed have not affected the activity of osteoblasts and osteoclasts. Gender differences in trace element indicate that pathways for trace element absorption and/or excretion vary between males and females. This issue however, requires further studies to expand our knowledge about that topic.

## Conflicts of Interest:

The authors declare no conflict of interest.

## Data Availability

The datasets generated during and analysed during the current study are available from the corresponding author on reasonable request.
